# Hypopion inverse secondaire à un accès de toux

**DOI:** 10.11604/pamj.2024.47.87.42517

**Published:** 2024-02-26

**Authors:** Taha Boutaj, Samira Tachfouti

**Affiliations:** 1 Ophthalmology Department “A”, Ibn Sina University Hospital (*Hôpital des Spécialités*), Mohammed V University, Rabat, Morocco

**Keywords:** Hypopion inverse, décollement de rétine, baisse de l’acuité visuelle, toux, Reverse hypopyon, retinal detachment, decreased visual acuity, cough

## Abstract

Silicone oil tamponade is one of the therapeutic methods used in complex retinal detachments caused by inferior and/or posterior dehiscence. However, it can cause complications, in particular, silicone oil migration into the anterior chamber or “invered hypopion”. We here report the case of a patient presenting to the Emergency Department with a sudden decrease in visual acuity in the right eye secondary to a coughing fit. Ophthalmological examination revealed uncorrected visual acuity reduced to perception of hand movements. Intraocular pressure was 27mmHg. The examination of ocular annexes was normal. Slit-lamp examination of the anterior segment revealed emulsified silicone oil suspended in the anterior chamber, hiding half of the pupil and forming ´reverse pseudo-hypopion´ or silicone oil “hyperpion”: the reflection produced by intraocular implant was visible. Fundus was inaccessible. The contralateral eye examination was normal. Treatment was based on anterior chamber washout and aspiration of silicone oil. The patient´s outcome was favorable: visual acuity improved to 3/10 with normal intraocular pressure.

## Image en médecine

Le tamponnement par huile de silicone est l´un des moyens thérapeutiques utilisés dans les décollements de la rétine complexes par déhiscences inférieures et/ou postérieures. Il est cependant source de complications notamment le passage en chambre antérieure ou « inversed hypopion ». Nous rapportons le cas d´un patient qui se présente aux urgences pour baisse de l´acuité visuelle brutale a l´œil droit secondaire à un accès de toux. L´examen ophtalmologique retrouve une acuité visuelle sans correction réduite aux mouvements des doigts. Le tonus oculaire était à 27mmHg. L´examen des annexes est sans particularités. L´examen à la lampe à fente du segment antérieur objective une émulsion de silicone en chambre antérieure en suspension cachant la moitié de la pupille formant un « pseudo-hypopion inverse » ou « hyperpion » siliconé. Le reflet de l´implant intra-oculaire était visible. Le fond d´œil était inaccessible. L´examen de l´œil controlatérale était normal. Le traitement consistait en lavage de la chambre antérieure avec aspiration de l´huile de silicone. L´évolution était favorable: l´AV est remontée à 3/10 avec un tonus normal.

**Figure 1:**
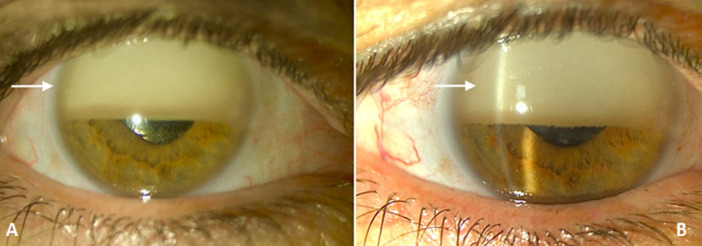
photographie à la lampe a fente: A) silicone en chambre antérieure en suspension formant un « pseudo-hypopion inverse »; B) la mise en place des deux fentes permet de voir le silicone piégé dans la chambre antérieure

